# Use of predicted vital status to improve survival analysis of multidrug-resistant tuberculosis cohorts

**DOI:** 10.1186/s12874-018-0637-0

**Published:** 2018-12-11

**Authors:** Meredith B. Brooks, Salmaan Keshavjee, Irina Gelmanova, Nataliya A. Zemlyanaya, Carole D. Mitnick, Justin Manjourides

**Affiliations:** 1 Department of Global Health and Social Medicine, Harvard Medical School, 641 Huntington Avenue, Boston, MA 02115 USA; 2 Department of Health Sciences, Northeastern University, 360 Huntington Avenue, Boston, MA 02115 USA; 3 Division of Global Health Equity, Brigham and Women’s Hospital, 75 Francis Street, Boston, MA 02115 USA; 4 Partners In Health, 800 Boylston Street, Suite 300, Boston, MA 02199 USA; 5 Partners In Health Russia, Moscow, Russian Federation

**Keywords:** Prediction models, Cox proportional hazards, Multidrug-resistant tuberculosis

## Abstract

**Background:**

Multidrug-resistant tuberculosis (MDR-TB) cohorts often lack long-term survival data, and are summarized instead by initial treatment outcomes. When using Cox proportional hazards models to analyze these cohorts, this leads to censoring subjects at the time of the initial treatment outcome, instead of them providing full survival data. This may violate the non-informative censoring assumption of the model and may produce biased effect estimates. To address this problem, we develop a tool to predict vital status at the end of a cohort period using the initial treatment outcome and assess its ability to reduce bias in treatment effect estimates.

**Methods:**

We derive and apply a logistic regression model to predict vital status at the end of the cohort period and modify the unobserved survival outcomes to better match the predicted survival experience of study subjects. We compare hazard ratio estimates for effect of an aggressive treatment regimen from Cox proportional hazards models using time to initial treatment outcome, predicted vital status, and true vital status at the end of the cohort period.

**Results:**

Models fit from initial treatment outcomes underestimate treatment effects by up to 22.1%, while using predicted vital status reduced this bias by 5.4%. Models utilizing the predicted vital status produce effect estimates consistently stronger and closer to the true treatment effect than estimates produced by models using the initial treatment outcome.

**Conclusions:**

Although studies often use initial treatment outcomes to estimate treatment effects, this may violate the non-informative censoring assumption of the Cox proportional hazards model and result in biased treatment effect estimates. Using predicted vital status at the end of the cohort period may reduce this bias in the analyses of MDR-TB treatment cohorts, yielding more accurate, and likely larger, treatment effect estimates. Further, these larger effect sizes can have downstream impacts on future study design by increasing power and reducing sample size needs.

## Background

Multidrug-resistant tuberculosis (MDR-TB) is caused by TB bacteria, *Mycobacterium tuberculosis,* being resistant to two powerful, first-line anti-TB drugs, rifampicin and isoniazid. Globally in 2016, an estimated 600,000 people were eligible for MDR-TB treatment, of which approximately 20% received care [1]. The latest available treatment outcome data showed that treatment success (including cure or treatment completed) occurred in only 54% of individuals, while 16% died, 8% had treatment failure, 15% were lost-to-follow-up, and 7% had no outcome information available [1]. Lack of safe and effective MDR-TB treatment is a major driving force behind MDR-TB as a global health problem [1]. The advent of two novel MDR-TB drugs [2, 3] and shortened regimens [4] offer opportunities for improved treatment access and outcomes. These developments further intensify the need for accurate estimation of treatment effectiveness. A common approach to assessing the effects of MDR-TB treatment on the risk of death is the Cox proportional hazards model [5], in part because of the variable treatment duration and for the ability to allow each individual to contribute survival time [6].

Recommendations for how to conduct an MDR-TB cohort analysis suggest that: a cohort should be developed based on the date of MDR-TB treatment initiation; analyses should be performed on all patients who receive treatment, regardless of duration; patients should be assigned the first of six mutually exclusive treatment outcomes that they experience; and patients should be followed for two years after the initial outcome to detect relapse [7]. However, while patients are usually followed by local programs from the time of treatment initiation until the first treatment outcome, information about longer survival is scarce due to the lack of resources in areas that experience the majority of the MDR-TB burden and the intensity of monitoring required for TB patients. When using limited data that is lacking information on survival after the initial treatment outcome, it is important to use the most efficient analysis methods to reduce potential bias in effect estimates.

Truncating patient survival times due to lack of follow-up data may bias treatment effect estimates when using proportional hazards regression due to violation of the non-informative censoring assumption of the model. This occurs when observations are censored from the data and assumed to be at equal risk of experiencing the event of interest (often death) as all at-risk individuals remaining in the cohort [8]. However, literature suggests that individuals who experience successful treatment outcomes (cure or treatment completion [7]) have a lower risk of death by the end of a defined cohort period (4%) compared to those who experience unsuccessful non-death treatment outcomes (treatment failure, treatment default, transfer out [7]; 60%) [9–15].

Literature exists on methods that can be used to adjust for informative censoring, such as inverse probability weighting (IPW) and competing risks regression. In the context of established MDR-TB treatment outcome definitions and standard practices for follow-up of patients, and where there is no administrative censoring due to the study ending, these methods have limitations. When using IPW, patients with an observed failure time are given weights according to the inverse probability of not being censored. These weights are estimated as a function of the observed outcomes and patient characteristics thought to predict censoring. IPW still relies on the assumption that subjects with observed outcomes are representative of the larger cohort [16–19]. When applied to MDR-TB cohort settings, the largest weights are assigned to subjects who are not censored, which are only those individuals who experience the event of interest: death. Because the vast majority of patients experience a non-death outcome, weighting observations in this manner does not approximate the characteristics of the larger cohort. Weighting non-censored observations does not resolve the need to estimate a differential risk of death based on the reason why an observation was censored, as patients who are censored due to experiencing successful treatment outcomes are likely to have very different survival trajectories compared to patients censored due to experiencing unsuccessful treatment outcomes.

The use of competing risks regression may also be inappropriate for use in these settings. A competing risk is an event that a patient experiences, other than the event of interest, which modifies the probability -- or completely precludes the occurrence -- of the event of interest [20]. Censoring, on the other hand, refers to an inability to observe the time at which an event occurs. Meeting the definition of an MDR-TB treatment outcome is not a new event--it is simply an intermediary outcome that is often used as a proxy for long-term outcomes because the event of interest is not yet observed due to follow-up being truncated. Therefore, we propose a more novel approach to overcome informative censoring in these cohort studies that accounts for the differential risk of death between individuals experiencing successful versus unsuccessful initial treatment outcomes.

The most accurate analysis would incorporate the true vital status at the end of the cohort period. When this is unavailable, however, due to follow-up ceasing after an initial treatment outcome occurs, it may be possible to improve treatment effect estimates by predicting the vital status of an individual at the end of the cohort period. This would account for a differential risk of death for individuals censored from the data due to experiencing different non-death treatment outcomes. Additionally, leveraging the initial treatment outcome to inform vital status may produce more accurate treatment effect estimates compared to censoring all observations from the data regardless of the reason follow-up was terminated.

Here, we seek to derive and validate a tool to predict vital status at the end of a cohort period and to assess how incorporation of the vital status into the Cox proportional hazards model affects bias in treatment effect estimates. Using initial treatment outcomes to inform estimates of the vital status at the end of the cohort period can provide useful information when modelling long-term survival. Models integrating the predicted vital status at the end of the cohort period were hypothesized to produce stronger and less biased effect estimates.

## Methods

### Study cohort

The study population is a cohort of consecutive patients with suspected or confirmed MDR-TB, who initiated treatment in Tomsk Oblast, Russian Federation between September 2000 and November 2004. Patients provided written informed consent prior to the initiation of TB therapy. TB providers collected data prospectively using standardize forms, and the study team reviewed medical charts in the Tomsk TB Control Program database to verify and complete these records. Data were entered into a dedicated electronic study database. More details about the enrollment and data collection methods for this cohort have been previously described [13, 15, 21, 22]. This cohort has patient data available up to six years after treatment initiation. These data include the date that the initial treatment outcome (routinely used as the outcome in MDR-TB cohort analyses) was assigned and date on which long-term vital status was assessed. This long-term vital status is rarely available in such cohorts and provides a unique opportunity to assess the true outcome of patients after MDR-TB treatment and the relationship between the initial treatment outcome and this outcome after longer follow-up. For this study, cohort participants were included if they had baseline MDR-TB, if data were available regarding treatment start and initial treatment outcome, and if vital status at the end of the study cohort period was discernable. Patients were classified as having MDR-TB if they had a culture positive for *M. tuberculosis* and drug susceptibility test results showing resistance to at least isoniazid and rifampin [22].

### Exposure variable definitions

The primary exposure of interest is receipt of an aggressive treatment regimen, which has previously been shown to improve treatment outcomes [22–25]. The aggressive treatment regimen is defined as a regimen containing at least five likely effective drugs based on previous treatment history and current drug resistance patterns during the intensive phase of treatment, followed by at least four likely effective drugs during the continuation phase of treatment [24–26]. A binary variable was used to classify each patient as ever or never having been exposed to an aggressive treatment regimen.

Other characteristics included are those previously identified as being risk factors for death [13, 21, 27, 28], including age, sex, alcohol abuse or dependence, presence of a comorbidity, prior treatment history, low body mass index (BMI), severe baseline clinical status, extra-pulmonary TB (EPTB), and extensively drug-resistant (XDR-) TB. Alcohol abuse or dependence was determined at baseline or at the time of the doctor prescribing medication. The presence of a baseline comorbidity (other than HIV) is defined as the presence of any of the following: diabetes mellitus, chronic renal insufficiency, seizure disorder, baseline hepatitis or transaminitis, or psychiatric disease. Prior treatment history is classified as more than two or less than or equal to two previous regimens. Low BMI is defined as < 20 kg/m^2^ for men and < 18.5 kg/m^2^ for women. Severe baseline clinical status is defined as respiratory insufficiency, hemoptysis, or sputum acid-fast bacilli smear (+++) at baseline [22]. XDR-TB is defined as the resistance to isoniazid, rifampin, any fluoroquinolone, and at least one of three second-line injectable drugs [29, 30].

### Outcome variable definitions

Standard MDR-TB treatment outcome definitions are used [22]. A successful treatment outcome encompasses treatment completion and cure. Unsuccessful treatment outcomes include treatment failure, all-cause mortality, default during treatment, or transfer out. Patients were followed from treatment start until the time when their first treatment outcome was observed. The cohort period is defined as the longest duration from treatment initiation until an initial treatment outcome. Vital status at the end of the cohort period is defined as whether a patient remained alive or had died prior to the end of the defined cohort period. The primary outcome is the time from treatment initiation until death.

### Statistical methods

To characterize the population, we describe demographic information, comorbidities, treatment characteristics, and treatment outcomes. Characteristics are quantified by the frequency and percent for categorical variables and means and standard deviations (SD), unless noted otherwise, for continuous variables. Selection bias is evaluated by assessing whether patient characteristics and treatment outcomes are statistically different between included and excluded participants through use of chi-square, Fishers exact test, or t-test.

Our analysis involves a two-step procedure. First, a logistic regression model is fit to predict the probability of survival at the end of the study period. Second, a Cox proportional hazards model is fit, incorporating recoded failure and censoring outcomes based on the vital status predicted in the logistic regression model.

- Step 1. Logistic regression model for long-term vital status:

A logistic regression model is used to predict the probability of survival at the end of the study period for each individual, *i*, who experienced a non-death initial treatment outcome. Vital status is modeled as a random variable, taking the value 1 with probability equal to the parameter *p*_*i*_, which is a function of the initial treatment outcome (*O*_*i*_) and patient characteristics (***X***_*i*_). The parameter *p*_*i*_ is estimated for each individual in the cohort.

Potential predictors eligible for the model include all combinations of the initial treatment outcomes and patient characteristics that may be associated with survival. Patient characteristics considered include those that are standardly collected globally, ensuring that the model may be applied to other TB cohorts in the future for external validation. For model derivation and internal validation, we use 10-fold cross-validation. Data are randomly divided into ten sets, the model is built on nine of these sets and then the performance of the model is measured on the remaining set. This is repeated until all ten data sets are used to test model performance. The model with the best performance is selected as the final model.

The primary means of comparing predictive models is the Bayesian Information Criterion (BIC) [31], for which lower values indicate better fit. We also use the *c*-statistic to assess model discrimination, the ability of the model to differentiate between individuals who died at the end of the study and those who did not. The larger the *c*-statistic, the better the model discriminates [32]. To assess model calibration, which describes the agreement between the predicted and observed risks, we compute the Hosmer-Lemeshow statistic [33]. We define good calibration as a Hosmer-Lemeshow statistic *p*-value greater than the type-one error rate of 0.05, indicating no evidence that the observed and predicted risks significantly differ.

A receiver operating characteristics (ROC) curve is used to select a probability threshold, through use of the Youden’s index, that maximizes the discriminative properties, including sensitivity, specificity, positive predictive value, and negative predictive value of the model. The Youden’s index is the vertical distance from the ROC diagonal chance line to each point on the curve and aims to minimize the false negative and positive rates [34]. Discriminatory property definitions are as follows: sensitivity is the probability of the model predicting survival at the end of the cohort period given the individual truly survived; specificity is the probability of the model predicting death prior to the end of the cohort period given the individual truly died; positive predictive value is the probability of surviving until the end of the cohort period given the model predicts survival; negative predictive value is the probability of dying prior to the end of the cohort period given the model predicts death. The probability threshold identified is used to assign each individual a vital status of alive (${\widehat{Y}}_i=1$) or dead (${\widehat{Y}}_i=0$) at the end of the study period (i.e., if the probability threshold is set at 0.85, then if ${\widehat{p}}_i$ > 0.85, ${\widehat{Y}}_i=1;$ if ${\widehat{p}}_i$< 0.85, ${\widehat{Y}}_i=0$).

- Step 2. Cox proportional hazard model:

To evaluate the bias introduced when survival information after the initial treatment outcome is lacking, we run two Cox proportional hazards models. Each model uses three different approaches for a total of six scenarios. Models 1 and 2 both assess the association between receipt of an aggressive treatment regimen and death. Model 1 assesses the univariate association, while Model 2 assesses the association while controlling for the covariates described earlier that were previously found to be associated with time to death.

The three approaches we use on each model are as follows:

- **Approach 1:** The first approach follows the *conventional censoring assumption* in which the event time for each individual is either the observed time to death or the time to the observed non-death treatment outcome, at which point censoring occurs.
- **Approach 2:** The second approach uses the *predicted vital status* at the end of the study period ($\widehat{Y}$). All individuals assigned a ${\widehat{Y}}_i=1$ are assumed to survive at least until the end of the cohort period and contribute full survival time during that period. All individuals assigned a ${\widehat{Y}}_i=0$ are assumed to be at equal risk of death as those at-risk individuals remaining in the cohort. These observations are censored at the time of an observed non-death treatment outcome.
- **Approach 3:** The third approach, the gold standard, utilizes the *true vital status* at the end of the study (*Y*_*i*_). Individual event times are either the time of death or time to the end of the cohort period, at which point all remaining, alive individuals are censored. This approach serves as the reference, against which values obtained from Approaches 1 and 2 are compared.

Estimated hazard ratios (HR) and 95% confidence intervals (CI) for the aggressive treatment regimen variable are presented for each model and approach. Relative change between the HRs for each model are calculated by comparing Approaches 1 and 2 to those from Approach 3. Relative to Approach 3, HRs closer to the null hypothesis of 1.0 underestimate the treatment effect, while HRs further from 1.0 overestimate the treatment effect. The magnitude and direction of the bias from Approaches 1 and 2 are assessed. Relative changes are compared to identify which approach produces the least biased effect estimates in relation to Approach 3.

SAS V9.4 (SAS Institute, Cary, NC) is used for all analyses.

Institutional Review Boards at Harvard School of Public Health (Boston, Massachusetts) and the Siberian State Medical University (Tomsk, Russia) approved the parent study. Secondary analysis was reviewed and declared exempt by the Institutional Review Board at Northeastern University (Boston, Massachusetts).

## Results

A total of 638 individuals with suspected or confirmed MDR-TB were consecutively enrolled during the study period. Of these, 614 individuals have confirmed MDR-TB by culture and drug susceptibility testing. The longest interval from treatment start until the initial treatment outcome is 1293 days, defining the duration of the study period. Among the 614 individuals, vital status at the end of the study period is unable to be ascertained for 167 (27.2%); these observations are excluded, leaving 447 eligible participants included in this analysis.

The mean age of the cohort is 35.9 (sd: 11.4) years, 81.2% are male; 53.0% have history of incarceration. Almost everyone (99.3%) has previously been treated for tuberculosis; many have had prior injectable (33.3%) and/or fluoroquinolone (15.8%) exposure. The mean number of previous tuberculosis treatments for the cohort is 2.1 (sd: 1.2), with one-third having greater than two previous treatments. Over half (62.8%) present with bilateral and cavitary disease on the baseline chest radiograph or with severe baseline clinical status (62.0%), and 4.9% present with baseline XDR-TB. Of the 447 included in the analysis, 82.6% receive an aggressive regimen at some point during MDR-TB treatment. Two-thirds of participants experience a successful initial treatment outcome while 6.7% died, 8.7% had treatment fail, and 17.4% defaulted on treatment. Full baseline characteristics for included participants are in Table 1.

**Table 1:** Baseline characteristics for 447 patients whose status at the end of the study period is known

Baseline characteristic/Outcome	Total (*N* = 447) *n*, %
Months on effective regimen (mean, sd)	11.6 (7.9)
Ever on effective regimen	369 (82.6)
Sociodemographic characteristics	
Age, years (mean, sd)	35.9 (11.4)
Female sex	84 (18.8)
Married (*n* = 434)	200 (46.1)
Unemployed (*n* = 445)	352 (79.1)
Current or previous incarceration	237 (53.0)
Alcohol abuse/dependence	194 (43.4)
Illicit drug use	79 (17.7)
Homelessness	16 (3.6)
Comorbidities	
HIV-positive (*n* = 446)	3 (0.7)
Diabetes mellitus (*n* = 446)	18 (4.0)
Comorbid condition	322 (72.0)
Prior TB treatment exposure	
Previously treated for TB	444 (99.3)
History of prior injectable exposure (*n* = 436)	145 (33.3)
History of prior fluoroquinolone exposure (*n* = 436)	69 (15.8)
History of prior default	16 (3.6)
Number of previous TB treatments (mean, sd)	2.1 (1.2)
> 2 previous TB treatment (*n* = 436)	141 (32.3)
Clinical indications of disease severity	
Bilateral and cavitary disease on baseline chest x-ray (*n* = 443)	278 (62.8)
Severe pulmonary disease on baseline chest x-ray	195 (43.6)
Low BMI at start of treatment (*n* = 446)	190 (42.6)
Severe baseline clinical status	277 (62.0)
Extrapulmonary disease (*n* = 381)	39 (10.2)
Previous TB-related surgery (*n* = 445)	50 (11.2)
Baseline XDR-TB	22 (4.9)
Initial treatment outcome	
Successful	299 (66.9)
Cure	280 (62.6)
Treatment Completion	19 (4.3)
Unsuccessful	148 (33.1)
Death	30 (6.7)
Treatment Failure	39 (8.7)
Default	78 (17.4)
Transfer Out	1 (0.2)

The 167 excluded participants are statistically, significantly different from those included in the following ways: fewer females, fewer married, more unemployed, more currently or previously incarcerated, fewer with severe baseline clinical status, more with EPTB, and more experienced an initial treatment outcome of default. Moving forward, the one participant who had a treatment outcome of transferred out was excluded from further analysis due to low sample size in that category.

### Predicting long term survival

Through 10-fold cross validation, we identify our final predictive model, which includes covariates for a successful initial treatment outcome, treatment failure, and age (centered):

Log $\left(\frac{p}{1-p}\right)$ = 2.56 + 2.46*Successful – 0.77*Failure – 0.04*Age

This final model is selected due to a combination of having a low BIC value (139.88), a high c-statistic (0.95), and a high Hosmer-Lemeshow statistic *p*-value (0.99). Table 2 shows top performing model characteristics using 10-fold cross validation, including the selected model (number 3).

**Table 2:** Model performance characteristics using 10-fold cross validation

Model covariates	BIC	Discrimination	Calibration
		C-statistic	Hosmer-Lemeshow test statistic (*p*-value)
1. Successful	157.72	0.90	0 (N/A)
2. Successful + failure	145.29	0.93	0 (1.00)
3. Successful + failure + age	139.88	0.95	1.28 (0.99)
4. Successful + failure + sex + age	136.91	0.95	2.05 (0.97)

**Fig. 1:**
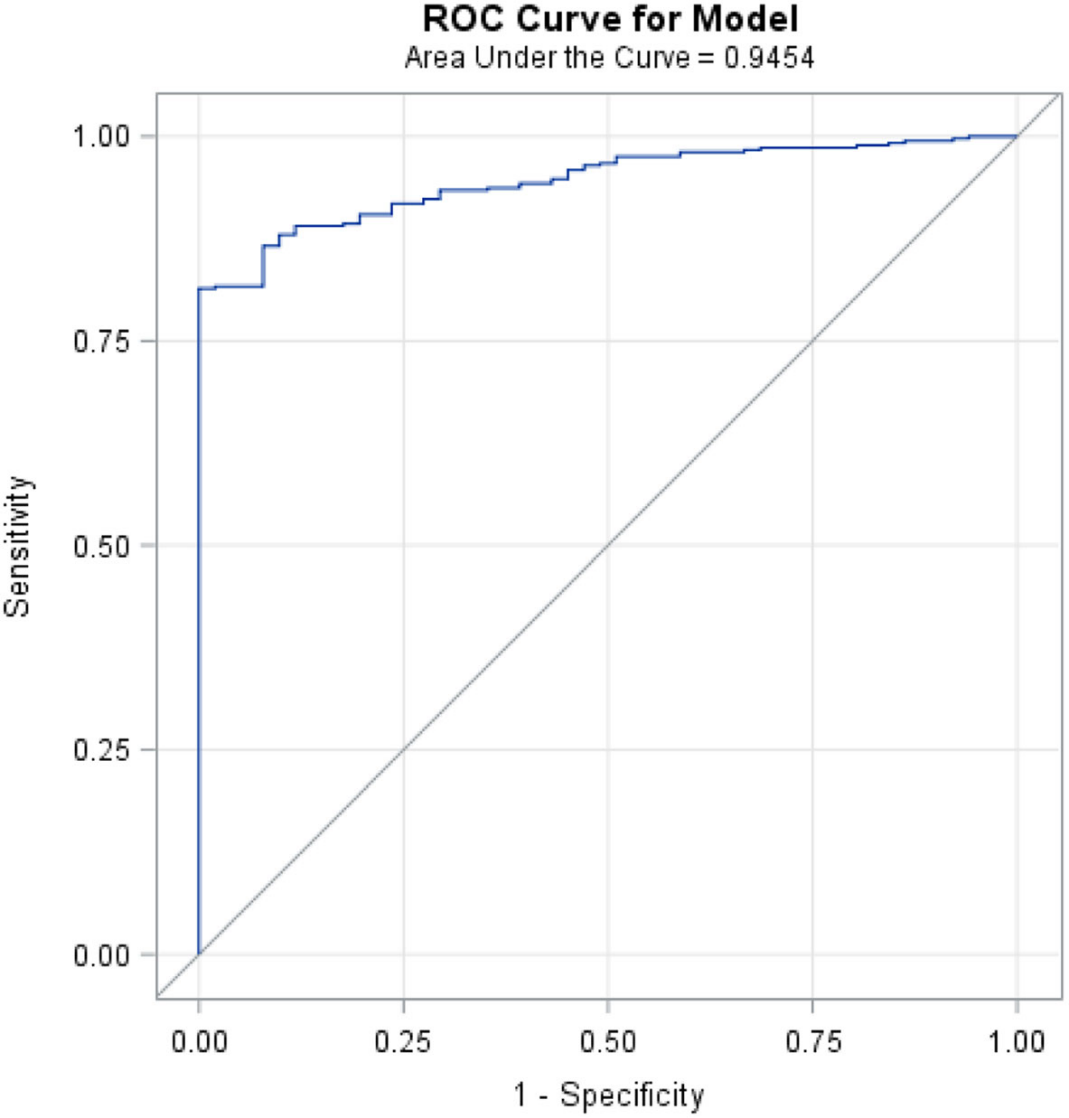
Receiver Operating Characteristics curve for final model selected

Using an ROC curve (see Fig. 1), we identify the best cutoff at 0.99, resulting in a sensitivity of 0.81 (95% CI: 0.77, 0.85), specificity of 1.00 (95% CI: 0.93, 1.00), positive predictive value of 1.00, and a negative predictive value of 0.43 (95% CI: 0.38, 0.48).

Using the predicted probabilities, 99.3% of subjects experiencing an initial successful non-death treatment outcome are estimated to remain alive at the end of the study period, which is close to the true outcome in which 99.7% remained alive (see Table 3). No patients who had experienced an initial unsuccessful non-death treatment outcome are predicted to stay alive, when in reality, 57.3% actually did. Over two-thirds of people defaulting treatment and one-third of people whose treatment failed truly remain alive at the end of the period.

**Table 3:** Distribution of predicted and actual vital status by initial treatment outcomes

Initial Treatment Outcome		Predicted vital status		Actual vital status	
*n* = 446 *n*, %		Alive *n*, % 297 (71.4%)*	Dead *n*,% 119 (28.6%)*	Alive *n*, % 365 (87.7%)*	Dead *n*,% 51 (12.3%)*
Successful	299 (67.0)	297 (99.3)	2 (0.7)	298 (99.7)	1 (0.3)
Cure	280 (62.8)	278 (99.3)	2 (0.7)	279 (99.6)	1 (0.4)
Treatment Completion	19 (4.3)	19 (100.0)	0 (0.0)	19 (100.0)	0 (0.0)
Unsuccessful	147 (33.2)	0 (0.0)	117 (100.0)^#^	67 (57.3) ^#^	50 (42.7) ^#^
Death	30 (6.7)	N/A	N/A	N/A	N/A
Treatment Failure	39 (8.7)	0 (0.0)	39 (100.0)	13 (33.3)	26 (66.7)
Default	78 (17.5)	0 (0.0)	78 (100.0)	54 (69.2)	24 (30.8)

*Out of 416 who experienced an initial non-death treatment outcome

^#^Out of 117 who experienced an initial non-death unsuccessful treatment outcome

Note: denominators for the alive and dead columns for the predicted and actual end-of-cohort treatment outcomes are the total from the initial treatment outcome column

In univariate analyses using Approach 1, receipt of an aggressive treatment regimen is protective against death (HR: 0.32; 95% CI: 0.15, 0.69). Compared to using Approach 3 (HR: 0.26; 95% CI: 0.17, 0.41), this results in a 22.1% relative change. The model using Approach 2 leads to a HR: 0.31 (95% CI: 0.14, 0.66), which results in a 16.7% relative change to the model using Approach 3. Approach 2 yields a reduction in the bias observed using Approach 1 by 5.4%. In multivariable analysis using Approach 1, receipt of an aggressive treatment regimen is still protective against death (HR: 0.24; 95% CI: 0.10, 0.54), resulting in a 6.3% change from the same model utilizing Approach 3 (HR: 0.22; 95% CI: 0.14, 0.36). The model using Approach 2 yields a HR: 0.23 (95% CI: 0.10, 0.52), resulting in a 3.2% relative change from Approach 3. Approach 2 yields a reduction in bias observed using Approach 1 by 3.1%. See Table 4 for more details.

**Table 4:** Change in effect estimates using varying approaches to handle censored observations

Model #	Covariate	Approach 1: Using initial treatment outcomes	Approach 2: Incorporating predicted vital status	Approach 3: Using actual vital status	Relative Change (Approach 1 & 3)	Relative Change (Approach 2 & 3)	Reduction in bias from using Approach 2 instead if Approach 1
		HR (95% CI)	HR (95% CI)	HR (95% CI)			
1	AR	0.32 (0.15, 0.69)*	0.31 (0.14, 0.66)*	0.26 (0.17, 0.41)**	22.1%	16.7%	5.4%
2		0.24 (0.10, 0.54)*	0.23 (0.10, 0.52)*	0.22 (0.14, 0.36)**	6.3%	3.2%	3.1%

*AR* Aggressive Regimen; *C*: Confidence Interval; *HR*: Hazard Ratio; *p-value: < 0.05; **p-value: < 0.0001

Model 1: Univariate

Model 2: Multivariable: receipt of an aggressive regimen, age, sex, alcohol abuse/dependence, baseline comorbidities, severe clinical status, XDR-TB [used covariates found significant in previous studies for which no data were missing as to not introduce imputation or missing data problems]

## Discussion

Compared to conventional methods of only following participants until they experience an initial treatment outcome, incorporating the predicted vital status at the end of the cohort period into Cox proportional hazards models can reduce bias in treatment effect estimates. Conventional methods utilizing time to the initial treatment outcome improperly censors survival times earlier due to lack of data about longer patient survival and leads to underestimation of the treatment effect by up to 22.1%. Models utilizing the predicted vital status at the end of the cohort period inform the amount of survival time an individual contributes to the model and leads to stronger effect estimates. This change is consistent across univariate and multivariable analyses.

Application of individual survival probabilities allows for distinction between successful and unsuccessful non-death treatment outcomes, which literature suggests result in different risk of survival at the end of a cohort period [9–15]. This differs from the conventional approach that effectively treats all censored observations as being at equal risk of death as those observations remaining in the cohort.

Our predictive model has good fit statistics, discrimination, and calibration. However, there are some limitations to this model. We observe a large false-negative misclassification rate. When the false-negatives produced from the predictive model are applied to the Cox proportional hazards model, we observe an underestimation of the true treatment effect because, instead of observations being accurately classified as ‘alive’ at the end of the cohort and contributing full survival time, they are classified as ‘dead’ and censored at the time of the initial treatment outcome. Reduction of the false-negative rate would produce stronger, more accurate treatment effect estimates. The one individual who was transferred out was excluded due to not having enough individuals in that category to estimate an accurate treatment effect. For populations with a larger percentage of individuals who are transferred out, it will be essential to better understand their long-term outcomes. Use of variables that are often strong risk factors for death in this population, such as alcoholism, could potentially strengthen the model. However, variables considered for use in the final model were only those that are universally collected so that the model can be validated in external cohorts.

In addition to model limitations, our study as a whole has several limitations that must be considered. As the goal of this study is to compare estimates among a naïve model, a predictive model, and a fully informed model (which requires end-of-cohort outcome knowledge), we excluded 167 patients with MDR-TB. They are different from those included, with a statistically significant higher proportion of men, unmarried, unemployed, with EPTB, and an initial treatment outcome of default. Significantly fewer had a severe baseline clinical status. Many of these patients were in the penitentiary sector, making them more likely to return home to a region outside of the study area after being released and, thus, more difficult to follow for long-term outcomes. If the differences between those included and excluded led to more deaths after the initial treatment outcome, bias may be introduced away from the null hypothesis, indicating a possible overestimation of the treatment effect.

Additionally, we only assess two options for using the predicted probabilities to inform the way in which observations are censored: censor at the time of the initial treatment outcome or censor at the end of the cohort period. Developing additional ways in which the observations are censored, such as at different time points after experiencing the initial treatment outcome, may be more realistic and produce more accurate treatment effect estimates. The predictive model is not validated in an independent cohort; however, it performed well when evaluated through 10-fold cross validation, which attempts to assess how the results will generalize to an independent data set.

## Conclusion

We found that using only the initial treatment outcome to analyze the treatment effect using Cox proportional hazards models underestimates the benefit of receiving an aggressive treatment regimen when compared to the fully informed model that incorporated the true long-term vital status. Incorporating predicted end-of-cohort vital status may reduce this bias in the analyses of MDR-TB treatment cohorts, allowing observation of larger and more accurate treatment effect sizes and, in turn, increasing study power.

We provide a simple-to-implement method to analyze data, which can potentially overcome the current limitation of MDR-TB cohorts lacking survival data past the initial treatment outcome. This method can allow researchers to estimate a range of potential effect estimates instead of one biased estimate. While the predictive model produces valid predictions for subjects from the underlying population, external validation is necessary before recommendation for use of the predictive model in other MDR-TB cohorts. While this approach may be used as a sensitivity analysis to predict the long-term effect of MDR-treatment, the most accurate treatment effect estimates can be obtained from following patients after their initial treatment outcome.

Improved accuracy of effect estimates is essential to guide MDR-TB treatment recommendations.

## Abbreviations

BIC: Bayesian information criterion
BMI: Body mass index
CI: Confidence interval
EPTB: Extra-pulmonary tuberculosis
HIV: Human immunodeficiency virus
HR: Hazard ratio
IPW: Inverse probability weighting
MDR-TB: Multidrug-resistant tuberculosis
ROC: Receiver operating characteristics
SD: Standard deviation
TB: Tuberculosis
XDR-TB: Extensively drug-resistant tuberculosis
